# Physical Localization of the Root-Knot Nematode (*Meloidogyne incognita*) Resistance Locus *Me7* in Pepper (*Capsicum annuum*)

**DOI:** 10.3389/fpls.2019.00886

**Published:** 2019-07-09

**Authors:** Amornrat Changkwian, Jelli Venkatesh, Joung-Ho Lee, Ji-Woong Han, Jin-Kyung Kwon, Muhammad Irfan Siddique, Abate Mekonnen Solomon, Gyung-Ja Choi, Eunji Kim, Yunhee Seo, Young-Ho Kim, Byoung-Cheorl Kang

**Affiliations:** ^1^Department of Plant Science, Plant Genomics and Breeding Institute and Vegetable Breeding Research Center, College of Agriculture and Life Sciences, Seoul National University, Seoul, South Korea; ^2^Research Center for Biobased Chemistry, Korea Research Institute of Chemical Technology, Daejoen, South Korea; ^3^Department of Agricultural Biotechnology and Research Institute of Agriculture and Life Sciences, Seoul National University, Seoul, South Korea

**Keywords:** co-segregating markers, dominant locus, fine mapping, *Me7*, NBS-LRR, resistance gene, root-knot nematode

## Abstract

The root-knot nematode (RKN) *Meloidogyne incognita* severely reduces yields of pepper (*Capsicum annuum*) worldwide. A single dominant locus, *Me7*, conferring RKN resistance was previously mapped on the long arm of pepper chromosome P9. In the present study, the *Me7* locus was fine mapped using an F_2_ population of 714 plants derived from a cross between the RKN-susceptible parent *C. annuum* ECW30R and the RKN-resistant parent *C. annuum* CM334. CM334 exhibits suppressed RKN juvenile movement, suppressed feeding site enlargement and significant reduction in gall formation compared with ECW30R. RKN resistance screening in the F_2_ population identified 558 resistant and 156 susceptible plants, which fit a 3:1 ratio confirming that this RKN resistance was controlled by a single dominant gene. Using the *C. annuum* CM334 reference genome and BAC library sequencing, fine mapping of *Me7* markers was performed. The *Me7* locus was delimited between two markers G21U3 and G43U3 covering a physical interval of approximately 394.7 kb on the CM334 chromosome P9. Nine markers co-segregated with the *Me7* gene. A cluster of 25 putative nucleotide-binding site and leucine-rich repeat (NBS-LRR)-type disease resistance genes were predicted in the delimited *Me7* region. We propose that RKN resistance in CM334 is mediated by one or more of these NBS-LRR class *R* genes. The *Me7*-linked markers identified here will facilitate marker-assisted selection (MAS) for RKN resistance in pepper breeding programs, as well as functional analysis of *Me7* candidate genes in *C. annuum*.

## Introduction

Root-knot nematodes (RKN; *Meloidogyne* spp.) are obligate phytopathogens causing significant economic losses in several crops including Solanaceae species (Eisenback and Triantaphyllou, 1991; Bernard et al., 2017). There are more than 98 *Meloidogyne* species (Jones et al., 2013). Four of these, *Meloidogyne arenaria*, *M. incognita*, *M. hapla*, and *M. javanica*, are widely distributed with diverse host ranges and cause considerable yield losses in many crops (Jones et al., 2013; Noling, 2014; Wu et al., 2018). Strategies to mitigate the RKN threat include biological and chemical control measures as well as the use of resistant cultivars/rootstocks. Due to the inefficiency of chemical applications, as well as increasing awareness of food safety and environmental concerns, the application of nematicides has been restricted. Since nematode-resistant cultivars provide an efficient and environmentally safe alternative to chemical measures, much effort has been devoted to identifying host resistance against RKN in cultivated or in wild species (Taylor and Sasser, 1978; Pegard et al., 2005).

Several dominant nematode resistance genes including *Mi1.1-2* and *Mi-9* have been reported in tomato (*Solanum lycopersicum*; Nombela et al., 2003; Seah et al., 2004; van der Vossen et al., 2005; Jablonska et al., 2007; Sanchez-Puerta and Masuelli, 2011; de Carvalho et al., 2015). In potato (*Solanum tuberosum*), the genes *Hero* and *Gpa2* confer resistance to *Globodera pallida* (white potato cyst nematode, van der Voort et al., 1999; van der Vossen et al., 2000; Ernst et al., 2002) and *Gro1-4* confers resistance to *Globodera rostochiensis* (yellow potato cyst nematode; Paal et al., 2004: Williamson and Kumar, 2006). In pepper, at least 10 dominant genes, *Me1* to *Me7*, *Mech1*, *Mech2* and *N* have been reported to confer resistance to *Meloidogyne* spp. (Wang and Bosland, 2006; Djian-Caporalino et al., 2007; Wang et al., 2009; Bucki et al., 2017). *Me1*, *Me3*, *Me7*, and *N* confer resistance to a wide range of RKNs, including *M. arenaria*, *M. javanica*, and *M. incognita*. *Mech1* and *Mech2* confer resistance to *M. chitwoodi*. These *R* genes originated from *Capsicum annuum* accessions PI 201234 (*Me1* and *Mech2*), PI 322719 (*Me3* and *Me4*), Criollo de Morelos 334 (CM334) (*Me7* and *Mech1*), and “Missisippi Nemaheart” (*N*) (Hare, 1957). Each of these genes have been deployed in pepper breeding programs (Fery et al., 1998; Djian-Caporalino et al., 2007; Wang et al., 2009, 2018; Fazari et al., 2012; Celik et al., 2016).

These RKN resistance genes, including *Me1*, *Me3*, *Me4*, *Me7*, *Mech1*, *Mech2*, and *N* have been mapped on the P9 chromosome and are clustered in a 28 cM genetic interval (Djian-Caporalino et al., 2001, 2007; Fazari et al., 2012; Uncu et al., 2015). *N* is linked to the *Me1* and *Me3* loci, but is not allelic to them. *Me3* and *Me7* were originally mapped to different loci 12.1 cM apart (Djian-Caporalino et al., 2007) on chromosome P9, but were later found to be allelic (Thies and Ariss, 2009; Djian-Caporalino et al., 2011; Fazari et al., 2012). Several molecular markers linked to the *Me1*, *Me3/Me7*, and *N* loci have been developed for marker-assisted selection (MAS) and are used in breeding programs (Wang et al., 2009, 2018; Fazari et al., 2012; Gisbert et al., 2013; Uncu et al., 2015; Guo et al., 2016). Despite extensive mapping studies, the molecular aspects of pepper RKN resistance genes remain largely unexplored.

Plant defense responses against RKN are often associated with gene-for-gene resistance (effector-triggered immunity). For instance, the well-known dominant *R* gene, *Mi-1* from *Solanum peruvianum* (Peruvian tomato), confers gene for gene resistance to some RKN *Meloidogyne* spp. *Mi-1* belongs to an *R* gene family that shares several canonical sequences and structural motifs of nucleotide-binding site (NBS) and leucine-rich repeat (LRR) domains (Milligan et al., 1998; Chen et al., 2007; Mao et al., 2015). The LRR domain induces hypersensitive response (HR)-like localized cell death (Hwang et al., 2000). Two RKN effectors, *MAP-1* from *M. incognita* and *Cg1* from *M. javanica*, have been suggested to be the cognate avirulence (*Avr*) genes of *Mi-1* (Gleason et al., 2008; Castagnone-Sereno et al., 2009). However, the interactions of these effectors with *Mi-1*, and *Mi-1*-mediated resistance, are not well understood. The potato cyst nematode RBP-1 and VAP1 effectors have been demonstrated to physically interact with their canonical R protein such as potato *Gpa2* and a tomato *Rcr3pim*/*Cf-2* and induce a foliar HR upon the transient co-expression (Sacco et al., 2009; Lozano-Torres et al., 2012).

CM334 is used by breeders as a source of resistance to a range of pathogens including several viruses, *Phytophthora capsaici* (chili pepper blight) and RKN (Palloix et al., 1990; Dogimont et al., 1996; Djian-Caporalino et al., 2007). Molecular markers have been developed to map the *Me7* locus (Djian-Caporalino et al., 2007; Fazari et al., 2012), with the closest markers, HM2, SCAR_PM6a, and SSCP_PM5 delimiting it to a 3.8 cM genetic interval (Fazari et al., 2012). However, the exact location of the *Me7* locus and the molecular basis of RKN resistance are poorly understood. To identify and clone the *Me7* gene, it is essential to construct a fine genetic map of the *Me7* locus and develop more closely linked markers.

Our aims were to construct a high-resolution map of the *Me7* locus and predict candidate genes for RKN resistance. A total of 28 markers, including high-resolution melting (HRM) and cleaved amplified polymorphic sequences (CAPS) markers, linked to RKN resistance were developed and used for fine mapping of the *Me7* locus using the F_2_ population derived from a cross between Early Calwonder 30R (ECW30R) and CM334. The tightly linked *Me7* markers we identified will be useful for marker-assisted selection for RKN resistance in pepper breeding programs.

## Materials and Methods

### Plant Materials

An F_2_ mapping population consisting of 714 plants, derived from a cross between *M. incognita* RKN-resistant CM334 and RKN-susceptible ECW30R lines from Horticultural Crops Breeding and Genetics Lab, Seoul National University, was used to fine-map the *Me7* locus. Phenotype screening of 504 of these F_2_ plants was performed at the Research Center for Biobased Chemistry, Korea Research Institute of Chemical Technology (KRICT), Daejoen, in 2014–2015 and phenotype screening of the other 210 F_2_ plants was performed at Seoul National University, Seoul, Korea, in 2015.

### Nematode Inoculation

*Meloidogyne incognita* race 1 was kindly provided by Prof. Y-HK (Clinical Plant Pathology and Nematology Laboratory, Seoul National University). *M. incognita* was propagated using susceptible tomato (*S. lycopersicum* cv. Micro-Tom). One-month old Micro-Tom plants were cultivated in pots with fresh commercial potting mixture (Hanarum, Minong Fertilizer, Korea) and sand in a 2:1 ratio were inoculated with 1,000 juvenile stage 2 (J2) of *M. incognita*. The inoculated plants were kept in the glasshouse condition with an average temperature of 26 ± 2°C. The nematode egg masses were collected from approximately 55-day-old infected susceptible tomato roots. Briefly, infected roots were cleaned with water and cut into 1 cm pieces, and then stirred in 1% NaOCl solution for 5 min (Coyne and Ross, 2014). The suspension was passed sequentially through a stack of sieves of 250, 180 and 25 μm, under running water, to remove any NaOCl residue. The eggs and J2 were captured on the 25 μm sieve with distilled water. The suspension with egg masses and J2 were filtered through a tissue paper that was placed on a Baermann funnel glass at room temperature (25°C) for three to four days. Thus, the J2 collected were counted under a light microscope. Four-leaf stage plants of testing populations were inoculated with 1,000 freshly hatched J2 *M. incognita* as mentioned above. Inoculated plants were kept in the glasshouse maintained at 26 ± 2°C.

### Nematode Resistance Screening

The resistance phenotype was evaluated at 45 days after inoculation (dai). For egg mass production and viability, four roots from each of the parental lines were uprooted and cleaned with tap water. Egg masses were handpicked from root system, and incubated in distilled water at room temperature for 2 h before observing under the magnifying microscope (Carl Zeiss, Thornwood, NY, United States). The root systems of each plant were examined, and the RKN resistance phenotype was scored using a root galling index (GI), rated on 0–4 scales, where 0 = 0–25%, 1 = 26–50%, 2 = 51–75%, 3 = 76-99%, and 4 = ≥ 100% (Baker, 1985). The percentage was calculated by dividing the total number of galls from each root system by the mean of the number of galls from susceptible ECW30R. The plants were classified as resistant when GI = 0 and as susceptible when GI ≥1, a classification modified from Seo et al. (2014).

### Analysis of Histological Responses

To study the histological response in the parental lines, infected roots as prepared above were harvested at 5, 10, and 15 dai for microscopic observation according to the procedure described by Seo et al. (2014). The specimens were sectioned by ultramicrotome (MT-X, RMC, Tucson, AZ, United States) to a thickness of 700 nm. The slides were stained with 1% toluidine blue O in 2% sodium tetraborate. The root systems of both parents were observed for HR by staining with fuchsin-acetic acid solution (Bybd et al, 1983). Specimens were analyzed using a Carl Zeiss microscope (Carl Zeiss, Thornwood, NY, United States).

### Genomic DNA Extraction

Total genomic DNA was extracted from young leaf tissues using the cetyltrimethylammonium bromide (CTAB) protocol (Kang et al., 2010). DNA concentration and quality were analyzed using a NanoDrop spectrophotometer (NanoDrop Technologies, Inc., Wilmington, DE, United States).

### Analysis of Previously Reported *Me* Linked Markers

Previously reported PCR-based markers including sequence characterized amplified region (SCAR), simple sequences repeats (SSR) and CAPS linked to the *Me1*, *Me3*, *Me7*, and *N* loci (Djian-Caporalino et al., 2007; Fazari et al., 2012; Guo et al., 2016) were amplified from the ECW30R and CM334 parents and sequenced at the National Instrumentation Center for Environmental Management (NICEM, Seoul National University, Korea). We analyzed these marker sequences for polymorphism and used BLAST to identify their physical positions in the CM334 genome version v.1.5 (scaffold) and v.1.55 (chromosome) (Kim et al., 2014). The SSR marker, CASSR37, linked to the *N* locus (Celik et al., 2016) and BAC-end markers PE43N9R, PE242G21R, PE11F6F, and PE25F15F, linked to the *Me3* locus derived from a double haploid pepper DH149 (Guo et al., 2016), were also used for marker development. The *Me*-loci and *N* linked markers are listed in Supplementary Table S1.

### Marker Development

To develop additional markers for fine mapping *Me7*, PCR primers were designed from the v.1.55 chromosome and v.1.5 scaffold versions of the *C. annuum* “CM334” genome (Kim et al., 2014). Amplified PCR products were sequenced at NICEM and Macrogen^1^ to detect polymorphisms. Identified SNPs were converted into HRM or CAPS markers listed in Supplementary Table S2.

### Genotype Analysis

The HRM assays were performed as described (Liu et al., 2016) with slight modifications in the PCR conditions as follows: 95°C for 4 min, followed by 50 cycles of denaturing at 95°C for 20 s, touchdown annealing from 60 to 53°C for 30 s, 72°C extension for 25 s, and then 25°C for 30 s. The HRM used 0.1°C increments between 65 and 90°C, with a Rotor-GeneTM 6000 thermocycler (Corbett Research, Sydney, Australia). The HRM curve profiles of homozygous parents and heterozygous F_1_ plants were used to assign the genotypes (Supplementary Figure S1). For the CAPS marker (18660) assay, PCR was performed as described by Jo et al. (2016). The digestion was performed in a reaction mixture containing 10 μl of PCR product, 2 μl of digestion buffer, 0.5 μl of *Msp*I and sterile distilled water in a total volume of 20 μl. Reactions were incubated for 3 h at 37°C. Digested PCR products were viewed using agarose gel electrophoresis (Supplementary Figure S2).

### BAC Library Screening

To expand the distal ends of scaffolds 1640 and 1578, markers from these scaffolds were used to screen a BAC library (Yoo et al., 2003). A three-step BAC screening procedure was described by Jo et al. (2016). BAC ends of 10 positive BAC clones were sequenced using the universal primers T_7_ and SP_6_. The BAC end sequences were then BLAST searched against the *C. annuum* genome databases^2^. The selected BAC611K18 clones were PacBio Pacific BioSciences) sequenced at Macrogen (see text footnote 1). The BAC sequence-derived SNPs and InDels were used for developing additional markers. The dot plot analysis of repeat sequences in the scaffold and BAC clones was performed using the NCBI server^3^.

### Comparative Analysis of the *Me7* Linked Markers and Physical Maps

To identify the most suitable sequence information to delimit the *Me7* locus, the total genome sequences from *C. annuum* “CM334” version v.1.55, v.1.6 (Kim et al., 2014, 2017) and UCD10X v1.0 (Hulse-Kemp et al., 2018) were used. CLC Main Workbench 8.1 (QIAGEN, Aarhus, Denmark) was used to obtain the physical position of the *Me7* linked markers, where map reads with ≥98% nucleotide similarity to the genomic sequence on chromosome P9 were used for comparative mapping.

### Fine Mapping and Gene Prediction

Genetic linkage analysis was performed using Carthagene ActiveTcl 8.4 (de Givry et al., 2005). The mapping distance was calculated using Kosambi’s mapping function with the LOD threshold set at 3.0 and distance threshold at 0.5. The genetic linkage map was drawn using the MapChart 2.2 tool (Voorrips, 2002). The *Me7* locus was initially mapped using 192 F_2_ plants and fine-mapped with 714 F_2_ plants. The physical map data for the *Me7* locus were retrieved from the pepper genome database version v.1.6^4^. The coding sequences (Annuum.v.2.0.CDS) from the genomic interval of the *Me7* locus were BLAST searched at the NCBI server (see text footnote 3) for identification of conserved domains and functional annotation.

## Results

### Analysis of Compatible and Incompatible Responses to RKN

**Figure 1:**
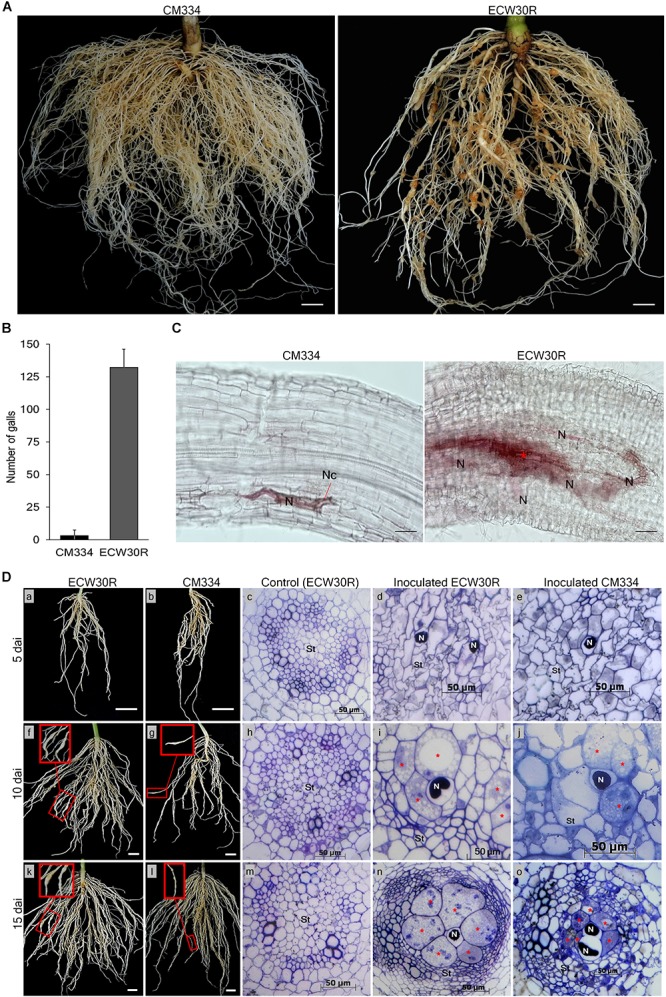
Comparison of resistant responses in the root systems of the parental lines. **(A)** A resistant CM334 and a susceptible ECW30R observed at 45 dai infected with *Meloidogyne incognita* J2. Bar = 1 cm. **(B)** Average number of root galls observed at 45 dai from 18 plants of each parent. Bar = standard deviation. **(C)** Resistance responses at 10 dai in CM334 and ECW30R root systems during juvenile penetration. Cell necrosis (Nc), nematode (N) and initiation of giant cell formation (red ^∗^). Bar = 100 μm. **(D)** Root systems and cross-sections of susceptible ECW30R and resistant CM334. The inoculated roots were harvested at 5, 10, and 15 dai. Pictures in red boxes pointing to galls development on the infected roots in close view (f, g, k, l), Bar = 1 cm. The root systems of ECW30R and CM334 and gall formation were observed at 5 (a–e), 10 (f–j), and 15 (k–o) dai. Cross-sections of inoculated ECW30R, CM334 and non-inoculated ECW30R (control) at 5 (c–e), 10 (h–j), and 15 (m–o) dai. Nematode (N), enlarged multinucleate giant cells (red ^∗^) and stele (St). Bar = 50 μm.

To know the resistance response to RKN in our experimental conditions, *M. incognita* gall formation and egg mass production were observed in resistant *C. annuum* “CM334” and susceptible *C. annuum* “ECW30R” at 45 dai (Figure 1A and Supplementary Figure S3). Viable egg masses with J2 were observed in both CM334 and ECW30R root systems (Supplementary Figures S3B,D,E), however, the root systems of CM334 formed only a few, small galls, while ECW30R roots had many, large galls (Figure 1A,B). We performed histological studies to investigate the intercellular resistance responses in the root systems. At 10 dai, no cell necrosis was noticed in ECW30R, whereas necrosis was observed in CM334 cells, as a result of a HR (Figure 1C), indicating that CM334 is highly resistant to *M. incognita*.

Cross-sections of the root system of RKN-infected plants revealed no morphological differences between susceptible ECW30R and resistant CM334 at five dai; however, juveniles of RKN were detected in both infected parents with no sign of feeding site formation (Figure 1D; a–e). At 10 dai, although giant cell development was initiated in both lines, the stele cells were slightly narrower and denser with enlarged feeding sites in ECW30R compared to CM334 (Figure 1D; f–j). At 15 dai, the infected cells in ECW30R grew much larger than in CM334 and caused increased compaction of cell layers in vascular tissues (Figure 1D; k–o). Overall, these results demonstrate that the RKN resistance of CM334 involves cell necrosis as well as the suppression of establishment and/or enlargement of feeding sites.

### Phenotyping and Inheritance Assay

The RKN resistance was evaluated at 45 dai. All tested CM334 and F_1_ plants were resistant to RKN, while all ECW30R plants were susceptible. Out of 714 F_2_ individuals, 558 plants showed resistance and 156 F_2_ plants showed susceptible phenotypes, which fit a segregation ratio of 3 resistant:1 susceptible, as expected for the inheritance of a single dominant gene (*X*^2^ = 3.782, *P* = 0.0518) (Table 1). These results are consistent with previous phenotyping studies (Djian-Caporalino et al., 2007; Fazari et al., 2012).

**Table 1:** Segregation analysis of RKN resistance in CM334, ECW30R, F_1_ and F_2_ mapping populations at 45 dai.

Parent lines and progenies	Number of plants			Expected ratio (R:S)	χ^2^ (df = 1)	*P = 0.05*
	Total	Resistant	Susceptible			
CM334	37	37	0	1:0		
ECW30R	55	0	55	0:1		
ECW30R × CM334 F_1_	30	30	0	1:0		
ECW30R × CM334 F_2_	714	558	156	3:1	3.782	0.0518

### Development of Markers Closely Linked to the *Me7* Locus

A high-density map for the *Me7* locus was developed using the *Me* loci and *N* locus-linked markers from previous studies (Supplementary Table S1), and new markers developed using genomic information from CM334 (Supplementary Table S2). Among the previously reported markers, only SCAR_PM6a, SCAR_PM6b (linked to *Me3*, *Me7*, and *N* loci) and CASSR37 (linked to *N* locus) were found to be polymorphic and therefore used for mapping *Me7* in this study. No recombinant were found for SCAR_PM6a and SCAR_PM6b, whereas three recombinants were detected for CASSR37 in a subset of the F_2_ population containing 192 individuals. These results indicate that the *N* and *Me7* loci are not allelic, which concurs with previous reports (Fazari et al., 2012; Celik et al., 2016). Four *Me3*-linked markers (PE43N9R, PE242G21R, PE11F6F, and PE25F15F) (Guo et al., 2016) were also tested; however, these markers were not polymorphic in our mapping population (Supplementary Table S1).

**Figure 2:**
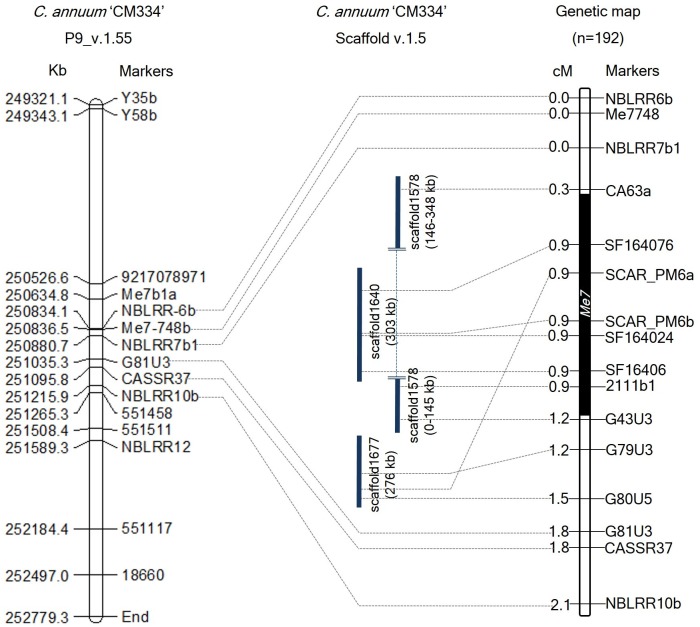
Comparative physical and genetic maps of the *Me7* locus developed from 192 F_2_ plants. The left panel indicates the physical map of the *Me7* linked markers on the “CM334” genome reference P9 (v.1.55). The right panel indicates the genetic map of the *Me7* locus. Six markers co-segregating with the *Me7* locus were detected on scaffolds (v.1.5). The *Me7* locus was delimited to a 0.9 cM region between the G43U3/G79U3 and CA63a markers. Physical and genetic distances between markers are indicated on the left side of the chromosome P9.

To develop additional markers, we BLAST searched these *Me3*, *Me7*, and *N* linked marker sequences against the *C. annuum* CM334 genome (v.1.55) and scaffold (v.1.5) (Kim et al., 2014), and identified their genomic positions on chromosome P9 and unassigned scaffold sequences. Using the genomic information of CM334 and scaffolds (1640, 1646, 1677, and 1578) containing SCAR_PM6a, SCAR_PM6b, CASSR37, and the four *Me3*-linked markers, we developed 21 HRM markers and one CAPS marker (Figure 2 and Supplementary Table S2). In 192 F_2_ individuals, four newly developed markers and two previous markers (SCAR_PM6a and SCAR_PM6b) co-segregated with the *Me7* locus (Figure 2) with a genetic interval of 0.9 cM on P9 (v.1.6) between G43U3, G79U3, and CA63a, which are located on scaffolds (1578, 1640, and 1677) (Figure 2 and Supplementary Table S2).

### BAC Library Screening, Sequence Analysis and Marker Development

**Figure 3:**
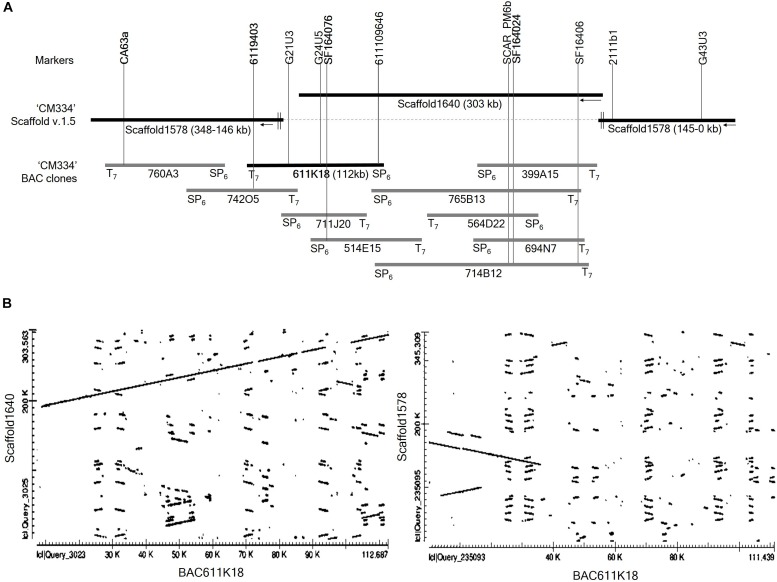
BAC library screening and alignment analysis. **(A)** Four markers (CA63a, 6119403, SF164076, and SF164024) derived from scaffold 1578, scaffold 1640 and BAC611K18 were used to screen the *Capsicum annuum* “CM334” BAC library (Yoo et al., 2003). BAC screening detected ten positive clones which were aligned on scaffold 1640 and 1578. **(B)** Dot plot analysis of full-length sequence of the BAC clone 611K18 revealed that 611K18 overlapped and bridged the gap between scaffold 1640 and 1578.

To extend the distal ends of scaffold 1640 and to identify the regions overlapping with scaffolds 1578, we performed BAC library screening using three markers: the CA63a marker from scaffold 1578 and the SF164076 and SF164024 markers from scaffold1640 (Figure 3A). BAC library screening with the marker CA63a detected the 760A3 clone, and both its BAC ends aligned to scaffold 1578 (Figure 3A). BAC screening with the marker SF164024 identified five clones, 399A15, 765B13, 564D22, 694N7, and 714B12. Of these, only 765B13 and 714B12 clones overlapped with 611K18 and 514E15 (Figure 3A). BAC library screening with the SF164076 marker detected three BAC clones, 711J20, 514E15, and 611K18. BAC end sequences of the 611K18 clone aligned to the sequence of the scaffolds 1640 and 1578 (Figure 3A). Therefore, we selected the 611K18 clone for full-length sequencing and developed five markers (Supplementary Table S2). Further BAC screening with the 6119403 marker developed from the 611K18 BAC clone detected a single clone, 742O5, which bridged the gap between the 760A3 and 611K18 clones (Figure 3A). Furthermore, the comparative dot plot analysis revealed an association between scaffolds 1640 and 1578 bridging and overlapping the BAC clones (Figure 3B). A total of five additional HRM markers (Supplementary Table S2) were produced using genomic information of BAC clone 611K18 and a flanking marker indicated by G21U3 for fine mapping of the *Me7* locus.

### Fine Mapping of the *Me7* Locus

**Figure 4:**
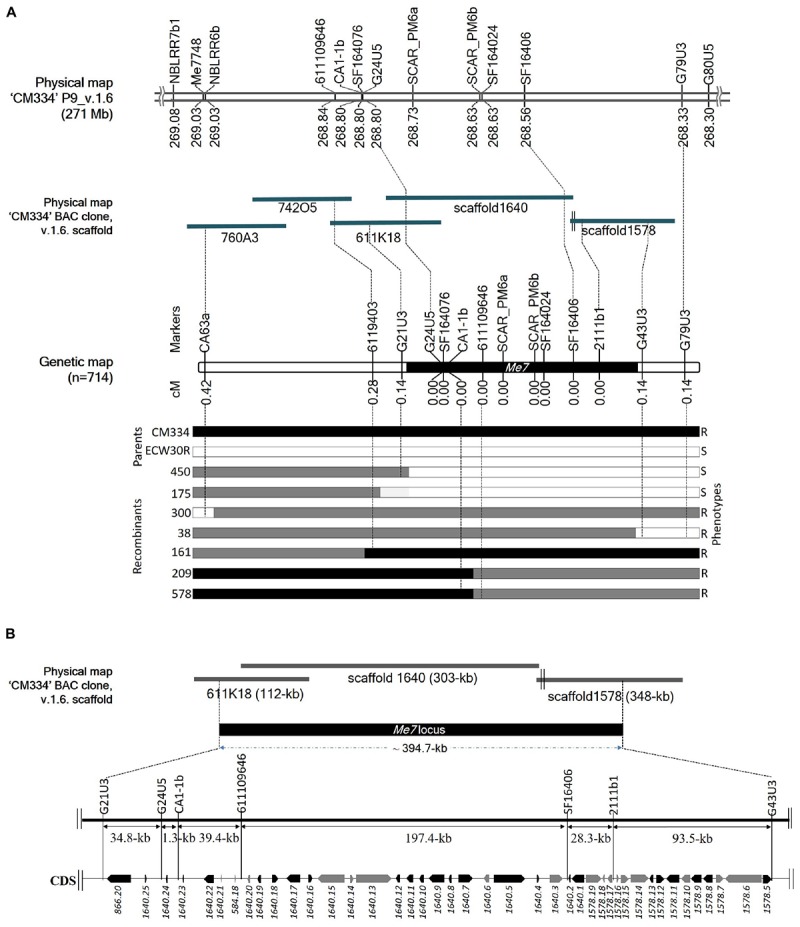
Fine mapping of the *Me7* locus. **(A)** The physical position of known and newly developed linked markers to the *Me7* locus on “CM334” chromosome P9, scaffold v.1.6 and BAC clones is shown. Fine mapping of the *Me7* locus showing the genetic map constructed from 714 F_2_ population and the introgression patterns. **(B)** The *Me7* locus was delimited to a ∼394.7 kb region (black area) between the G21U3 and G43U5 markers covered by the *Capsicum annuum* “CM334” BAC contig 611K18, scaffold 1640 and 1578. Gene prediction and BLAST search analysis against Annuum.v.2.0.CDS showed 42 genes in the target region. The genes colored in black are NBS-LRR type and genes in gray indicate non- NBS-LRR genes.

Initial mapping of the *Me7* locus in an F_2_ population consisting of 192 individuals, placed SCAR_PM6a and SCAR_PM6b 0 cM from the *Me7* locus. We used 31 markers, including 28 newly developed ones, to narrow the target region and we increased the size of the mapping population to 714 plants (Figure 4A and Supplementary Tables S1, S2). This fine mapping analysis produced seven recombinant plants (Figure 4A; 38, 161, 175, 209, 300, 450, and 578); one recombinant for each of G21U3, G43U3, and G79U3, two recombinants for 6119403 and three recombinants for CA63a (Figure 4A). Three markers (CA63a, 6119403, and G21U3) were placed on the one side of the *Me7* locus, whereas two markers (G43U3 and G79U3) were placed on the other side (Figure 4A). Nine markers including seven newly developed markers and two reference markers (SCAR_PM6a and SCAR_PM6b) co-segregated with the *Me7* locus. The *Me7* locus was thus delimited to a 0.28 cM region with an interval of approximately 394.7 kb between G21U3 and G43U3 covered by BAC clone 611K18 and scaffolds 1640 and 1578 (Figure 4A,B).

### Prediction of the *Me7* Gene Candidates and Coding Sequence (CDS) Analysis

A total of 42 CDS were identified from the ∼394.7 kb target region (Figure 4B) covered by scaffold 1640, scaffold 1578, and BAC611K18. BLAST analysis of the corresponding predicted proteins revealed that 25 belonged to the NBS-LRR family and the other 17 were unknown hypothetical proteins (Table 2). The NBS-LRR candidates contained four different types of other conserved domains, including the potato virus X resistance protein (RX), ATPases associated with a variety of cellular activities (AAA), reverse transcriptase (RNA-dependent DNA polymerase) group 2 (RVT_2), or reverse transcriptase (RT) (Table 2). Among the 25 predicted NBS-LRR family genes, many of them are truncated, lack both Toll/interleukin-1 receptor (TIR) and coiled-coil (CC) motifs. Only six NBS-LRR family genes (*1578.8*, *1578.12*, *1640.1*, *1640.7*, *1640.17*, and *1640.22*) were found to be related to CC-NBS-LRR (CNL) type *R* gene class. Furthermore, these NBS-LRR proteins shared sequence similarity with putative late blight resistance proteins including R1A-3, R1A-4, R1A-10, R1B-8, R1B-14, R1B-16, and R1B17 (Table 2). We thus consider these tightly linked NBS-LRR class resistance (*R*) genes as strong candidates to underly the RKN resistance conferred by the *Me7* locus.

**Table 2:** Predicted genes from the ∼394.7 kb *Me7* locus by BLAST alignment from the predicted CDS Annuum.v.2.0.CDS.

Gene ID	CDS size (bp)	NCBI conserved domain hits	NCBI Blastn hits: *Capsicum annuum*	Query cover (%)	*E*-value	Identity (%)	GenBank ID
*866.20*	2634	PLN00113, STKc_IRAK, RNase_HI_RT_Ty1	PREDICTED: probable LRR receptor-like serine/threonine-protein kinase At3g47570 (LOC107856496), mRNA	85%	0	92%	XM_016701500.1
*1640.25*	333	NBS-LRR	PREDICTED: putative late blight resistance protein homolog R1B-14 (LOC107840730), mRNA	94	2.00E-68	82	XM_016684605.1
*1640.24*	333	NBS-LRR	PREDICTED: putative late blight resistance protein homolog R1B-14 (LOC107840730), mRNA	94	2.00E-68	82	XM_016684605.1
			PREDICTED: putative late blight resistance protein homolog R1B-16 (LOC107841050), mRNA	92	8.00E-63	81	XM_016685060.1
			PREDICTED: putative late blight resistance protein homolog R1A-10 (LOC107840989), transcript variant X1, mRNA	78	1.00E-61	83	XM_016684974.1
*1640.23*	336	NBS-LRR	PREDICTED: putative late blight resistance protein homolog R1B-16 (LOC107876981), mRNA	10	3.00E-156	96	XM_016723782.1
*1640.22^∗^*	1908	RX-CC, NBS-LRR	PREDICTED: putative late blight resistance protein homolog R1A-3 (LOC107877461), mRNA	99	0	99	XM_016724105.1
			PREDICTED: putative late blight resistance protein homolog R1B-16 (LOC107852551), transcript variant X2, mRNA	99	0	99	XM_016697588.1
			PREDICTED: putative late blight resistance protein homolog R1B-16 (LOC107852551), transcript variant X1, mRNA	99	0.00E+00	99	XM_016697587.1
*1640.21*	192	PLN02365	–	–	–	–	–
*584.18*	771	RNase_HI_RT_Ty1	PREDICTED: serine/threonine-protein kinase At5g01020-like (LOC107877496), transcript variant X3, mRNA	18%	5.00E-53	93%	XM_016724148.1
			PREDICTED: uncharacterized mitochondrial protein AtMg00810-like (LOC107845361), partial mRNA	13%	7.00E-47	100%	XM_016689638.1
*1640.20*	525	–	PREDICTED: putative late blight resistance protein homolog R1A-3 (LOC107854603), mRNA	99	9.00E-177	88	XM_016699613.1
			PREDICTED: putative late blight resistance protein homolog R1A-10 (LOC107840990), transcript variant X2, mRNA	99	1.00E-174	88	XM_016684976.1
*1640.19*	669	NBS-LRR	PREDICTED: putative late blight resistance protein homolog R1A-3 (LOC107841011), transcript variant X5, mRNA	89	0	94	XM_016684997.1
			PREDICTED: putative late blight resistance protein homolog R1A-3 (LOC107841011), transcript variant X4, mRNA	89	0	94	XM_016684996.1
*1640.18*	1251	NBS-LRR	PREDICTED: putative late blight resistance protein homolog R1B-8 (LOC107852210), mRNA	67	0	94	XM_016697263.1
			PREDICTED: putative late blight resistance protein homolog R1A-3 (LOC107841054), mRNA	28	1.00E-113	94	XM_016685063.1
*1640.17*	2835	RX-CC, NBS-LRR, NBS	PREDICTED: putative late blight resistance protein homolog R1A-3 (LOC107854603), mRNA	94	0	88	XM_016699613.1
			PREDICTED: putative late blight resistance protein homolog R1B-16 (LOC107840770), transcript variant X2, mRNA	94	0	88	XM_016684673.1
*1640.16^∗^*	816	NBS-LRR	PREDICTED: putative late blight resistance protein homolog R1B-16 (LOC107852538), mRNA	100	0	100	XM_016697576.1
*1640.15*	975	CYCLIN, Cyclin_N2	PREDICTED: uncharacterized LOC107844017 (LOC107844017), ncRNA	18	1.00E-84	98	XR_001666539.1
			PREDICTED: uncharacterized LOC107852165 (LOC107852165), transcript variant X3, ncRNA	47	2.00E-83	98	XR_001669306.1
*1640.14*	636	TIP49	PREDICTED: uncharacterized LOC107852165 (LOC107852165), transcript variant X3, ncRNA	99	0	99	XR_001669306.1
*1640.13*	678	–	PREDICTED: putative late blight resistance protein homolog R1B-16 (LOC107852540), mRNA	87	0	100	XM_016697578.1
*1640.12*	636	NBS-LRR	PREDICTED: late blight resistance protein R1-A-like (LOC107852560), transcript variant X2, mRNA	99	2.00E-156	98	XM_016697593.1
*1640.11*	1245	AAA, NBS-LRR	PREDICTED: late blight resistance protein R1-A-like (LOC107852560), transcript variant X1, mRNA	76	0	99	XM_016697592.1
*1640.10^∗^*	867	NBS-LRR	PREDICTED: putative late blight resistance protein homolog R1B-16 (LOC107840741), transcript variant X4, mRNA	29	6.00E-68	85	XM_016684638.1
*1640.9*	960	NBS-LRR	PREDICTED: putative late blight resistance protein homolog R1B-8 (LOC107852210), mRNA	42	1.00E-159	92	XM_016697263.1
*1640.8*	450	NBS-LRR	–	–	–	–	–
*1640.7*	2805	RX-CC, NBS-LRR	PREDICTED: putative late blight resistance protein homolog R1B-16 (LOC107852559), mRNA	96	0	98	XM_016697590.1
*1640.6*	333	HemeO	PREDICTED: heme oxygenase 1, chloroplastic-like (LOC107840774), mRNA	98	2.00E-152	96	XM_016684676.1
*1640.5*	810	NBS-LRR	PREDICTED: putative late blight resistance protein homolog R1B-16 (LOC107852561), transcript variant X2, mRNA	84	0	100	XM_016697595.1
*1640.4*	480	NBS-LRR	PREDICTED: putative late blight resistance protein homolog R1B-16 (LOC107852561), transcript variant X2, mRNA	100	0	99	XM_016697595.1
*1640.3*	840	–	PREDICTED: putative late blight resistance protein homolog R1B-16 (LOC107852561), transcript variant X2, mRNA	67	0	100	XM_016697595.1
*1640.2*	363	NBS-LRR	PREDICTED: putative late blight resistance protein homolog R1B-16 (LOC107840741), transcript variant X4, mRNA	30	2.00E-33	90	XM_016684638.1
*1640.1^∗^*	2274	RX-CC, NBS-LRR	PREDICTED: putative late blight resistance protein homolog R1A-3 (LOC107840762), mRNA	99	0	94	XM_016684661.1
*1578.19*	1542	HAD_like	PREDICTED: calcium-transporting ATPase, endoplasmic reticulum-type (LOC107839342), transcript variant X7, mRNA	77	0	96	XM_016682783.1
*1578.18*	447	PPR_2	PREDICTED: pentatricopeptide repeat-containing protein At5g08510-like (LOC107839343), mRNA	100	0	93	XM_016682784.1
*1578.17*	813	PPR_2	PREDICTED: pentatricopeptide repeat-containing protein At5g08510-like (LOC107839343), mRNA	68	0	95	XM_016682784.1
*1578.16*	444	–	PREDICTED: probable membrane-associated kinase regulator 6 (LOC107879248), mRNA	99	1.00E-163	90	XM_016726325.1
			PREDICTED: uncharacterized LOC107877479 (LOC107877479), transcript variant X7, ncRNA	52	2.00E-112	98	XR_001676380.1
*1578.15*	426	TIP49	PREDICTED: uncharacterized LOC107877479 (LOC107877479), transcript variant X7, ncRNA	88	7.00E-107	99	XR_001676380.1
*1578.14*	1317	CYCLIN, Pox_A6	PREDICTED: uncharacterized LOC107844017 (LOC107844017), ncRNA	22	4.00E-144	98	XR_001666539.1
*1578.13*	1128	NBS-LRR	PREDICTED: putative late blight resistance protein homolog R1B-16 (LOC107852212), mRNA	95	0	94	XM_016697264.1
*1578.12*	2667	RX-CC, NBS-LRR	PREDICTED: putative late blight resistance protein homolog R1B-16 (LOC107852562), transcript variant X1, mRNA	61	0	98	XM_016697596.1
*1578.11*	2622	AAA, NBS-LRR	PREDICTED: putative late blight resistance protein homolog R1B-8 (LOC107852210), mRNA	48	0	91	XM_016697263.1
			PREDICTED: putative late blight resistance protein homolog R1B-16 (LOC107852531), mRNA	25	0	97	XM_016697573.1
*1578.10^∗^*	1875	–	PREDICTED: putative late blight resistance protein homolog R1B-17 (LOC107852564), mRNA	100	0	100	XM_016697598.1
*1578.9*	1218	RVT_2, NBS-LRR	PREDICTED: putative late blight resistance protein homolog R1A-10 (LOC107852537), mRNA	84	0	100	XM_016697575.1
			PREDICTED: putative late blight resistance protein homolog R1B-16 (LOC107877460), mRNA	75	0	98	XM_016724104.1
*1578.8*	3441	RT, RX-CC, NBS-LRR, NBS	PREDICTED: putative late blight resistance protein homolog R1B-17 (LOC107852536), mRNA	49	0	99	XM_016697574.1
*1578.7*	909	PPR_2	PREDICTED: pentatricopeptide repeat-containing protein At5g08510-like (LOC107839343), mRNA	100	1.00E-158	93	XM_016682784.1
*1578.6*	2289	HAD_like	PREDICTED: calcium-transporting ATPase, endoplasmic reticulum-type (LOC107839342), transcript variant X7, mRNA	57	0	95	XM_016682783.1
*1578.5*	1377	NBS-LRR	PREDICTED: putative late blight resistance protein homolog R1A-4 (LOC107852554), mRNA	46	0	100	XM_016697589.1

^∗^*Partial CDS*.

## Discussion

Understanding the molecular and genetic mechanisms of RKN resistance is of utmost importance for the development of RKN-resistant pepper lines. Although the RKN resistance gene *Me7* from CM334 was previously mapped to a 3.8 cM genetic interval, its identity remained unknown. The primary aim of current study was to develop a high-resolution map of the *Me7* locus and predict candidate genes. We were able to narrow down the *Me7* locus to an approximately 394.7 kb region covered by the BAC clone 611K18 and unassigned scaffolds 1640 and 1578 (Kim et al., 2014) containing 42 predicted genes. Among these, 25 genes were predicted to belong to the NBS-LRR family of disease resistance genes. The *Me1* gene (CA09g16830), which is a homolog of the resistance protein R1A-3 (Wang et al., 2018) belong to the CNL type *R* gene was found to be located approximately 3.3 and 1.1 Mb from the *Me7* locus based on the CM334 v.1.6 (Kim et al., 2017) and UCDv1.0 (Hulse-Kemp et al., 2018) reference genomes, respectively (Supplementary Figure S4).

During phenotype screening, we found galls and egg masses of *M. incognita* in the root system of the resistant CM334 variety, in contrast to the previous studies of Pegard et al. (2005), who did not observe egg masses at 6 weeks after inoculation. In the present study, inoculations were done with 1000 J2 instead of 300 to 600 J2 used in the previous works (Djian-Caporalino et al., 1999, 2001, 2007; Pegard et al., 2005), which may explain the development of egg masses. Similarly, occasional egg masses were also reported to be produced on HDA149 pepper line, carrying *Me3* (an allele of *Me7*) after inoculation with >1,000 J2 or ≥3,000 eggs (Castagnone-Sereno et al., 1996; Thies and Ariss, 2009). Thus, evidence from this study and previous research suggest that high inoculum densities (J2 ≥ 1,000 or eggs ≥3,000), has potential for resistant breaking in populations carrying *Me (s)* genes, such as *Me3* and *Me7* (Castagnone-Sereno et al., 1996; Thies and Ariss, 2009; Djian-Caporalino et al., 2011).

The ability of the nematode to overcome the plants early HR is highly correlates with RKN virulence levels. For instance, in *Me3*-resistant PM687 and HDA149 peppers formation of giant cells are no longer be prevented if cell necrosis in the outer barriers (epidermis and root cortex) has been overcome (Castagnone-Sereno et al., 1996). By contrast, in *Me1* carrying peppers, PM217 and HDA330 defense reactions could takes place even later infection stages, at near or inside the vascular tissues and thus blocks nematode development (Castagnone-Sereno et al., 1996). In the present study, CM334 root tissue cross-sections suggested that it has at least two kinds of resistance to *Meloidogyne* spp.: firstly, resistance that supresses penetration by J2 and secondly, resistance that blocks development after penetration (Pegard et al., 2005). Furthermore, in our histological study, we also observed inhibition of feeding site enlargement and minimal vascular tissue damage in CM334 resistant line, suggesting an extra layer of resistance in CM334 that suppressed feeding site enlargement.

To fine-map the *Me7* locus in pepper, we utilized information from previous mapping studies (Fazari et al., 2012; Celik et al., 2016) in combination with comparative mapping results (Supplementary Figure S4) from different versions of pepper genome databases (Kim et al., 2014, 2017; Hulse-Kemp et al., 2018). However, gaps in the genome sequence data and assembly errors due to multiple homologs with high nucleotide similarity hindered marker development and subsequent mapping of the *Me7* locus. BAC library screening has previously been integrated to assist marker development in complicated repetitive genomic regions or unreliable genome sequence assemblies (Jo et al., 2016). Here, by screening a BAC library, we were able to fill gaps between scaffold sequences (1640 and 1578) with a bridge from the BAC611K18 clone, which aided the development of tightly linked *Me7* markers. Furthermore, the release of the latest pepper genome update (version v.1.6, Kim et al., 2017) also partially allowed the integration of clone 611K18 and unassigned scaffolds 1640 and 1677 into chromosome P9.

A number of genes conferring resistance to parasitic plant nematodes encode NBS-LRR domains. Notably, the cyst nematode-resistance gene *Hero* from tomato (Ernst et al., 2002), *Gro1-4* (Williamson and Kumar, 2006) and *Gpa2* (van der Voort et al., 1999; Sacco et al., 2009) from potato and the RKN-resistance genes, *Mi-1*, *2*, and *9* from tomato (Milligan et al., 1998; Seah et al., 2004; Jablonska et al., 2007), pepper RKN-resistance genes, *CaMi* which is homologous to *Mi1.2* (Chen et al., 2007) and *Me1*, the homolog of putative late blight resistance protein R1A-3 gene (Wang et al., 2018) and *Ma* from *Prunus* spp. (Claverie et al., 2011) are reported to encode NBS-LRR proteins (Milligan et al., 1998; van der Vossen et al., 2000; Paal et al., 2004; Claverie et al., 2011). Similarly, we identified a cluster of 25 genes that were predicted to belong to the NBS-LRR *R* gene family, and which are strong candidates to be the *Me7* gene. Several plant NBS-LRR class *R* genes cluster at specific genomic locations due to tandem and/or segmental duplications in the course of evolution (Hulbert et al., 2001; Leister, 2004; Huang et al., 2005; McDowell and Simon, 2006). For instance, dominant *R* genes such as *Ph*-*3* from tomato, *R1* from potato, and *RpsUN1*, *RpsUN2*, and *Rpg1-b* from soybean (Ballvora et al., 2002; Ashfield et al., 2003; Zhang et al., 2014; Li et al., 2016) belong to NBS-LRR domain-encoding complex *R* gene clusters. *R* gene clusters could be sources of novel *R* genes, as such clusters enhance the possibility of structural and copy number variation arising through various molecular mechanisms (Michelmore and Meyers, 1998; Torii, 2004; Nagy and Bennetzen, 2008). The RKN resistance-related *R* gene clusters identified in this study could be associated with RKN resistance in CM334. However, further studies are required to reveal the specific NBS-LRR gene encoding the *Me7* gene.

Cloning of the *R* genes would be a key step toward unraveling the molecular mechanisms involved in disease resistance. However, the complexity of a large genome size as well as assembly errors at the *Me7* locus hinders identification of the *Me7* gene (Jupe et al., 2012). Recent developments such as resistance gene enrichment sequencing (RenSeq) and Single-Molecule Real Time (SMART) RenSeq techniques hold great promise for yielding higher sequencing read depth for individual genes, and aid in accurate identification of sequence variations in plants with large complex genomes (Jupe et al., 2012, 2013; Witek et al., 2016). RenSeq allows genome complexity reduction and enrichment of NBS-LRR type plant disease resistance genes in fully or partially assembled genomes (Jupe et al., 2012, 2013; Witek et al., 2016) and has been utilized for reannotation and identification of NBS-LRR *R* genes of Solanaceae plants such as tomato, potato, and tobacco. For instance, in potato, the number of NB-LRR identified increased from 438 to 755 through the RenSeq approach (Jupe et al., 2013). In pepper, studies to accurately determine the pepper *R* genes are now underway (we are currently undertaking RenSeq studies), which will further aid in the accurate prediction and functional characterization of the NB-LRR *R* genes identified in the present study.

## Conclusion

In conclusion, we successfully delimited the *Me7* locus to a genomic region of approximately 394.7 kb. We developed a total of 28 markers linked to the *Me7* locus and identified nine markers co-segregating with RKN resistance. Notably, the newly delimited *Me7* target region included a cluster of 25 NBS-LRR class candidate *R* genes. The tightly linked *Me7* markers will facilitate MAS in pepper breeding programs as well as assisting with mapping of other nematode-resistant genes in *C. annuum*.

## Data Availability

All datasets generated for this study are included in the manuscript and/or the Supplementary Files.

## Author Contributions

AC led the design of this study, including marker development, genetic and physical mapping, and BAC library screening. AC and G-JC developed mapping populations. J-WH and MS conducted DNA extraction. J-HL and AS were involved in the bioinformatics analysis. Y-HK provided *M. incognita* race 1. AC, J-KK, EK, and YS conducted nematode extraction and histological studies. AC and JV analyzed histological images and drafted and revised the manuscript. B-CK participated in the conception of this study, in discussions, and in the revision of the manuscript. All the authors have read and approved the final version of this manuscript.

## Conflict of Interest Statement

The authors declare that the research was conducted in the absence of any commercial or financial relationships that could be construed as a potential conflict of interest.

## Footnotes

1 https://dna.macrogen.com/eng/

2 http://passport.pepper.snu.ac.kr/?t=CAB

3 http://blast.ncbi.nlm.nih.gov/Blast.cgi

4 http://peppergenome.snu.ac.kr/download.php

## Supplementary Material

FIGURE S1 Example of HRM analysis of the *Me7* locus-linked markers. The analysis curves included HRM curves and melt curves. Blue, resistant (homozygous); red, susceptible; and green, resistant (heterozygous).

FIGURE S2 Restriction patterns of the CAPS marker, 18660. *Msp*I digested PCR product from susceptible and resistant lines show a clear bands of 560 and 808 bp, respectively. Heterozygous resistant lines show both 560 and 808 bp bands. L indicate 1 kb DNA molecular size marker.

FIGURE S3 The RKN egg masses produced in CM334 and ECW30R roots, imaged at 45 dai. Eggs are exuded egg mass on the outside of the female RKN on roots of CM334 **(A,B)** and ECW30R **(C,D)**. The egg mass from both CM334 **(B)** and ECW30R **(D)**. Different developmental stages of RKN, from egg to hatching of J2 are indicated **(E)**. e, egg; em, egg mass; r, root; rg, root gall; J0-1, egg developing undergo to J1. Bar = 0.5 mm.

FIGURE S4 Comparative analysis of the *Me7* linked markers on P9 from three genome references. The physical position of the *Me7* linked markers on “CM334” P9 version v.1.55, v.1.6 and UCD10X v1.0 (Kim et al., 2014, 2017; Hulse-Kemp et al., 2018) is shown. BLAST alignment was performed using CLC Main Workbench 8.1 (QIAGEN, Aarhus, Denmark), where map reads with ≥98% nucleotide similarity are shown. Markers that co-segregated with the *Me7* locus in the screening with 714 F_2_ individuals are represented in green.

TABLE S1 *Me* loci and *N* linked markers from previous studies.

TABLE S2 Newly developed markers used in the *Me7* locus mapping.
