# Supplementary figures and images for: Physical Localization of the Root-Knot Nematode (Meloidogyne incognita) Resistance Locus Me7 in Pepper (Capsicum annuum)
Source: Front Plant Sci. 2019 Jul 9;10:886. doi: 10.3389/fpls.2019.00886 (PMC6629957; doi:10.3389/fpls.2019.00886)

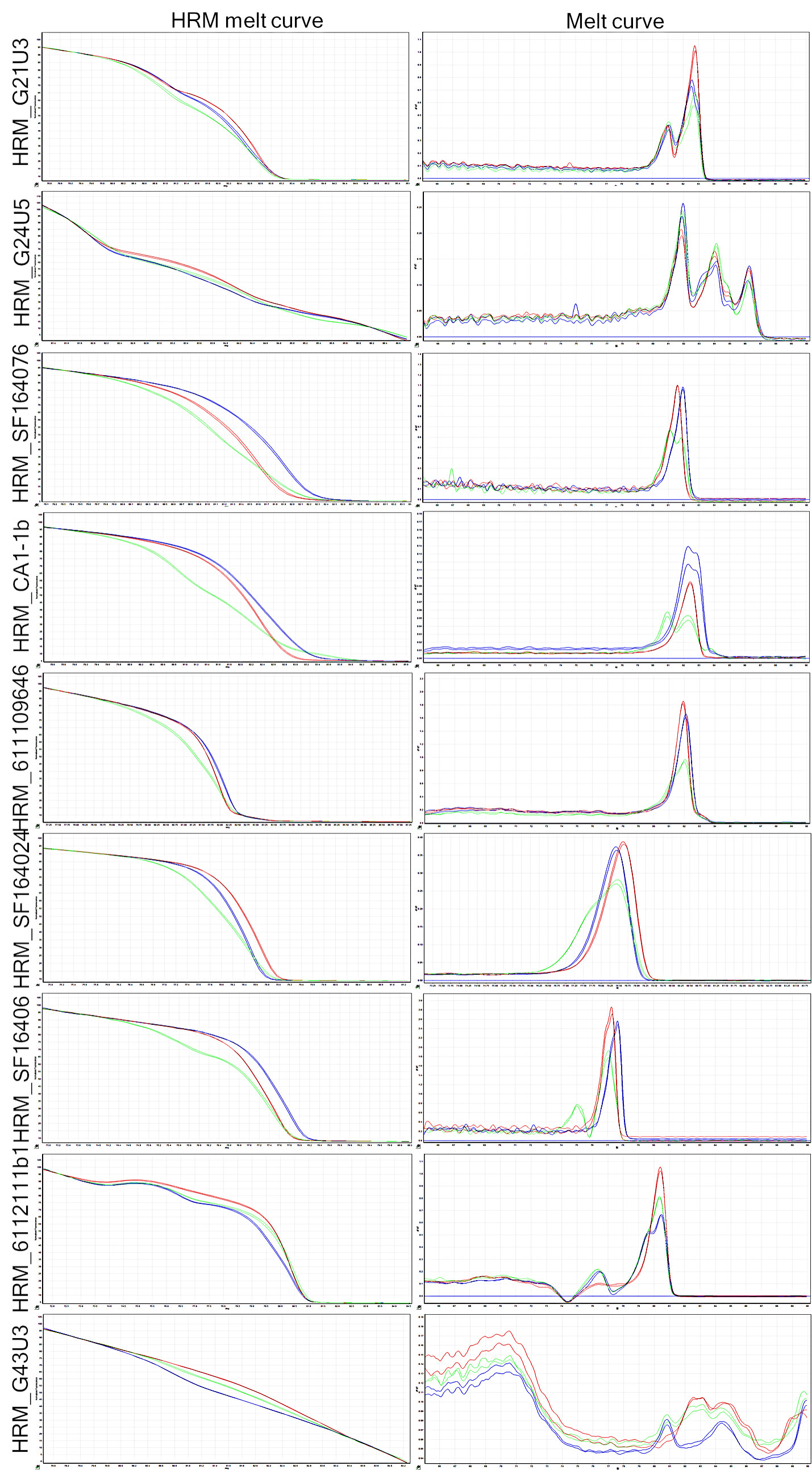

Supplement: FIGURE S1 — Example of HRM analysis of the Me7 locus-linked markers. The analysis curves included HRM curves and melt curves. Blue, resistant (homozygous); red, susceptible; and green, resistant (heterozygous). [file Image_1.JPEG]

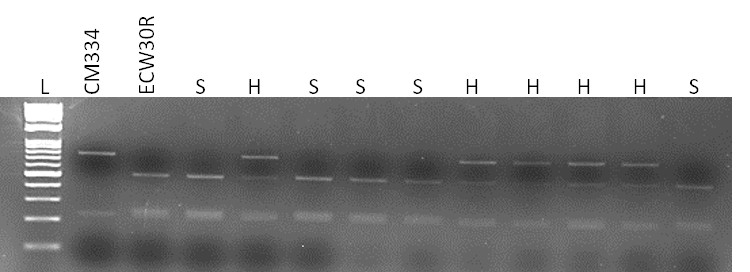

Supplement: FIGURE S2 — Restriction patterns of the CAPS marker, 18660. MspI digested PCR product from susceptible and resistant lines show a clear bands of 560 and 808 bp, respectively. Heterozygous resistant lines show both 560 and 808 bp bands. L indicate 1 kb DNA molecular size marker. [file Image_2.JPEG]

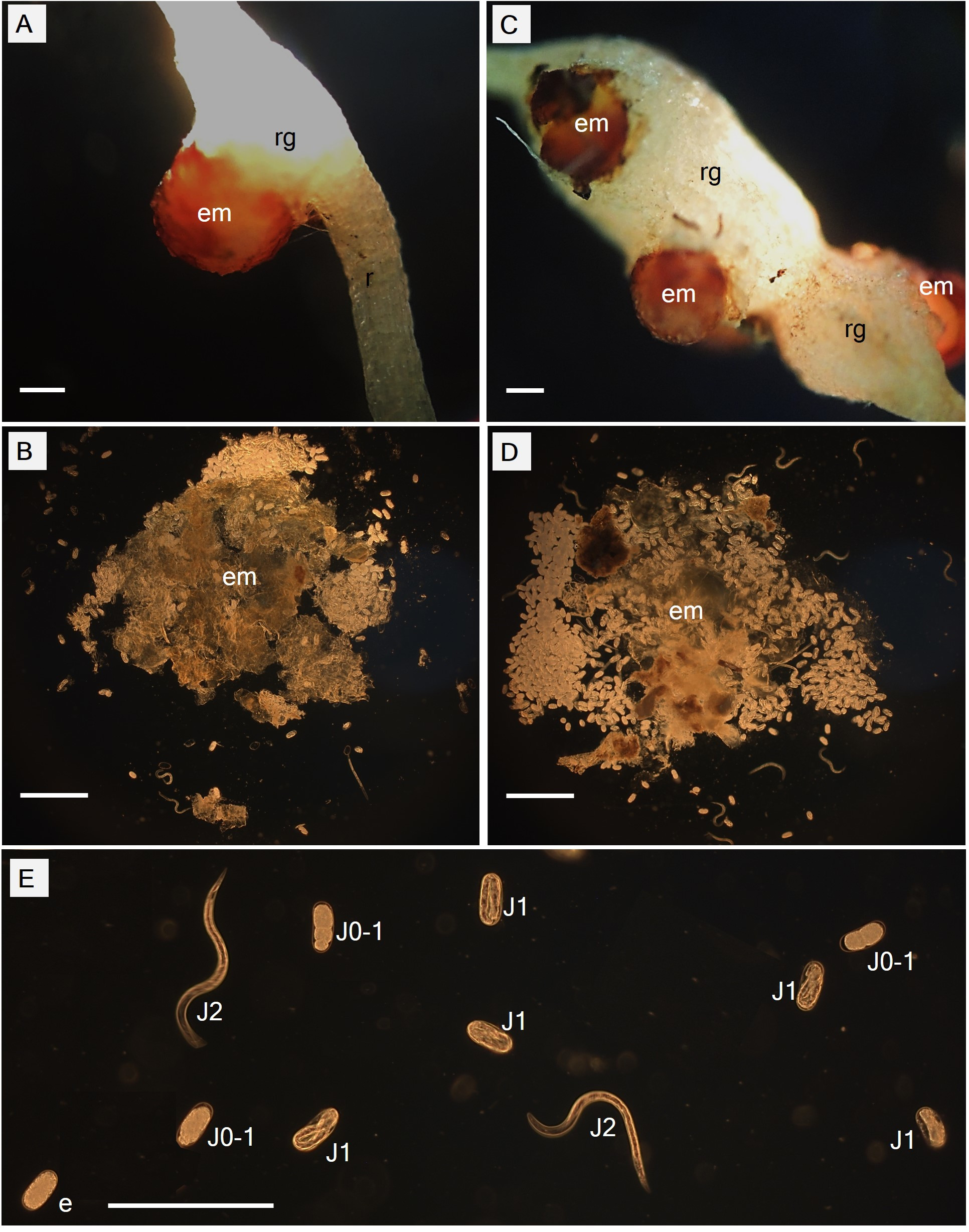

Supplement: FIGURE S3 — The RKN egg masses produced in CM334 and ECW30R roots, imaged at 45 dai. Eggs are exuded egg mass on the outside of the female RKN on roots of CM334 (A,B) and ECW30R (C,D). The egg mass from both CM334 (B) and ECW30R (D). Different developmental stages of RKN, from egg to hatching of J2 are indicated (E). e, egg; em, egg mass; r, root; rg, root gall; J0-1, egg developing undergo to J1. Bar = 0.5 mm. [file Image_3.JPEG]

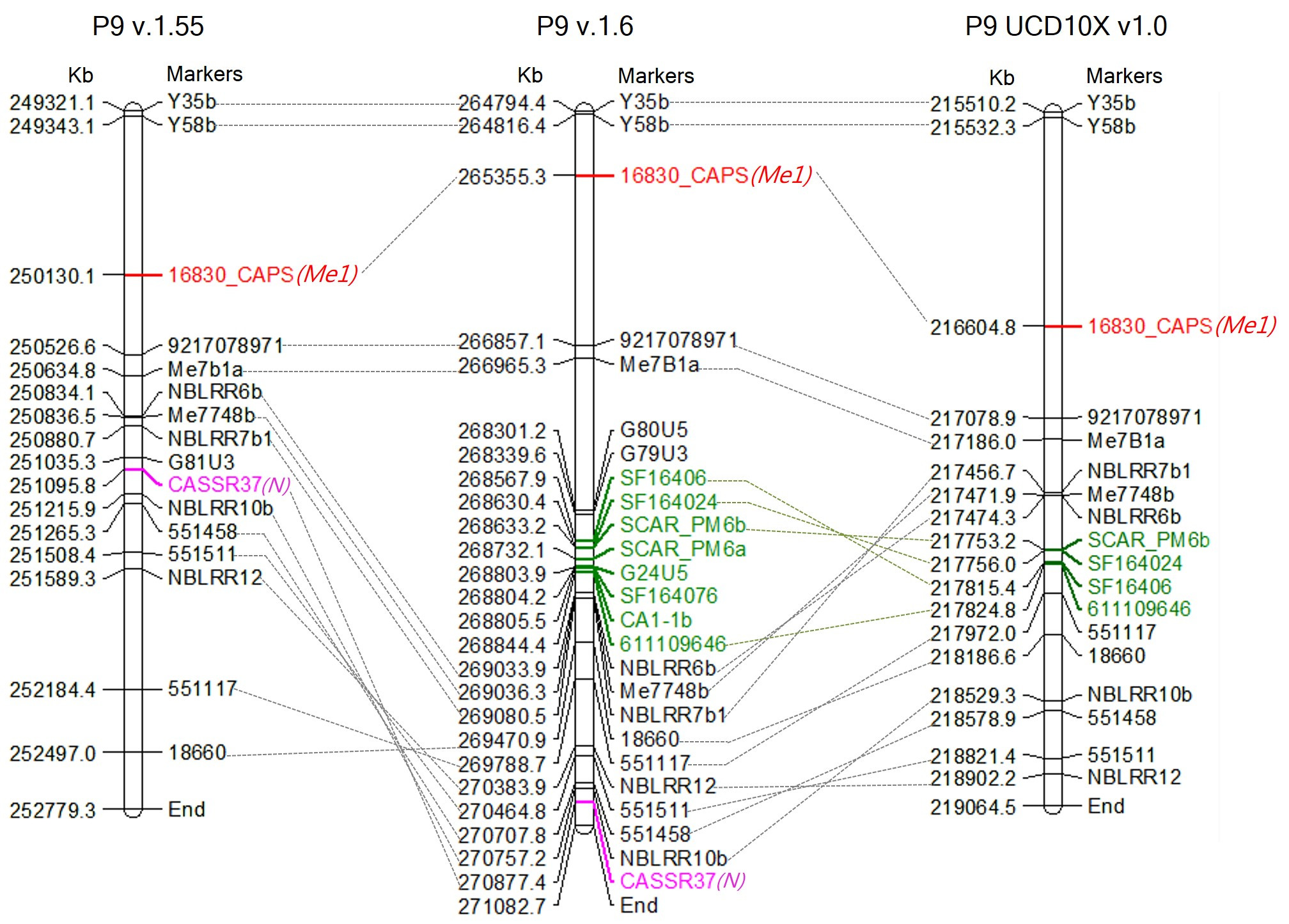

Supplement: FIGURE S4 — Comparative analysis of the Me7 linked markers on P9 from three genome references. The physical position of the Me7 linked markers on “CM334” P9 version v.1.55, v.1.6 and UCD10X v1.0 (Kim et al., 2014, 2017; Hulse-Kemp et al., 2018) is shown. BLAST alignment was performed using CLC Main Workbench 8.1 (QIAGEN, Aarhus, Denmark), where map reads with ≥98% nucleotide similarity are shown. Markers that co-segregated with the Me7 locus in the screening with 714 F2 individuals are represented in green. [file Image_4.JPEG]
